# Chronic Myeloid Leukemia Progressing to Early T-Cell Precursor Lymphoblastic Blast Phase Following Treatment Default: An Integrated Morphologic, Immunophenotypic, Cytogenetic, and Molecular Case Report

**DOI:** 10.7759/cureus.117154

**Published:** 2026-09-29

**Authors:** Sushma Cherukuru, Surya Prakashrao, Sachin J, Mukkanteswararao Kasaragadda, Sambasivarao Patibandla, Kiran K, Shalini Singh

**Affiliations:** 1 Pathology, American Institute of Pathology and Laboratory Sciences Pvt. Ltd. (AMPATH), Hyderabad, IND; 2 Cytogenetics, American Institute of Pathology and Laboratory Sciences Pvt. Ltd. (AMPATH), Hyderabad, IND; 3 Hematology, Citizens Specialty Hospitals, Hyderabad, IND; 4 Genetics, American Institute of Pathology and Laboratory Sciences Pvt. Ltd. (AMPATH), Hyderabad, IND

**Keywords:** abl1 f317l, bcr::abl1, blast phase, chronic myeloid leukemia, clonal evolution, dasatinib, del(7q), early t-cell precursor, etp-all, trisomy 8

## Abstract

Chronic myeloid leukemia (CML) is a BCR::ABL1-positive myeloid neoplasm characterized by the Philadelphia chromosome resulting from t(9;22)(q34;q11.2). Although tyrosine kinase inhibitors (TKIs) have substantially improved outcomes in chronic-phase CML, progression to blast phase remains an important feature of advanced disease. The majority of CML blast-phase cases demonstrate myeloid or precursor B-cell differentiation, whereas T-lineage transformation is uncommon. Early T-cell precursor acute lymphoblastic leukemia (ETP-ALL) represents an immature T-lineage neoplasm characterized by expression of cytoplasmic CD3 and CD7, weak or absent expression of conventional mature T-cell antigens, and frequent expression of myeloid or stem-cell-associated markers.

A 35-year-old man with a known diagnosis of CML in chronic phase, receiving dasatinib and reported to be a frequent treatment defaulter, presented with blast transformation. Peripheral blood examination demonstrated marked leukocytosis, anemia, and thrombocytopenia, with 64% circulating blasts. The blasts were large, with scant cytoplasm and pleomorphic nuclei. Flow cytometric immunophenotyping identified an abnormal blast population comprising approximately 82% of viable events. The blasts expressed CD34, HLA-DR, CD123, cytoplasmic CD3, CD5 (dim), CD7, CD13, and CD33, with variable expression of CD117 and CD56. They were negative for surface CD3, CD1a, CD4, CD8, B-lineage markers, TCR-γδ, and myeloperoxidase. The overall immunophenotype was consistent with ETP-ALL. Cytogenetic analysis demonstrated 47,XY,del(7)(q21),+8,t(9;22)(q34;q11.2), indicating the presence of the Philadelphia chromosome together with trisomy 8 and del(7q). Molecular analysis detected an ABL1 p.Phe317Leu (F317L) kinase-domain mutation.

This case represents a rare instance of CML blast phase with an ETP lymphoblastic phenotype and demonstrates the coexistence of an ABL1 F317L kinase-domain mutation and additional cytogenetic abnormalities, including trisomy 8 and del(7q). The case emphasizes the importance of integrating the clinical history with morphologic, flow cytometric, cytogenetic, and molecular findings when an immature T-cell leukemia is identified in a patient with established CML.

## Introduction

Chronic myeloid leukemia (CML) is a clonal hematopoietic neoplasm characterized by the Philadelphia chromosome, which results from a reciprocal translocation between ABL1 at 9q34 and BCR at 22q11.2. The resultant BCR::ABL1 fusion protein has constitutive tyrosine kinase activity and plays a central role in the pathogenesis of CML [1,2].

The introduction of BCR::ABL1-directed tyrosine kinase inhibitors (TKIs) has markedly changed the natural history of CML, with most patients diagnosed in the chronic phase achieving prolonged survival when appropriately treated and monitored [1]. Nevertheless, progression to accelerated or blast phase remains a clinically important event and is associated with aggressive disease biology and poor outcomes [2,3].

CML blast phase is most commonly myeloid or precursor B-lymphoid in phenotype, whereas T-cell or natural killer-cell differentiation is distinctly uncommon [4-7]. Historical reports have demonstrated that blasts arising during CML blast crisis may exhibit early T-cell characteristics, providing evidence for the multilineage differentiation potential of the CML-associated hematopoietic clone [4-7].

Early T-cell precursor acute lymphoblastic leukemia (ETP-ALL) is a rare immature T-lineage leukemia characterized by a distinctive immunophenotype, classically comprising cytoplasmic CD3 positivity, CD7 positivity, weak or absent CD5 expression, absence of CD1a and CD8, and expression of stem-cell and/or myeloid-associated antigens [8].

The occurrence of an ETP-ALL phenotype during CML blast transformation is extremely rare. Earlier case reports documented early T-cell differentiation in Ph-positive CML blast crisis [4-7], and Senapati et al. subsequently reported an ETP-type lymphoid blast crisis as a rare form of CML transformation [9]. More recently, Shuyu et al. described three patients with CML blast phase presenting with an ETP-ALL phenotype and highlighted the diagnostic challenge of distinguishing transformed CML from de novo Philadelphia chromosome-positive ETP-ALL [3].

We describe a case of CML progressing to blast phase with an ETP lymphoblastic phenotype, associated with an ABL1 F317L kinase-domain mutation and additional cytogenetic abnormalities, including trisomy 8 and del(7q).

## Case presentation

A 35-year-old man with a known diagnosis of CML in chronic phase was receiving treatment with dasatinib. The patient was reported to be a frequent treatment defaulter. He subsequently developed blast transformation and underwent peripheral blood examination, flow cytometric immunophenotyping, ABL1 kinase-domain mutation analysis, and conventional cytogenetic evaluation.

The diagnostic investigations were performed on peripheral blood collected in July 2026.

Cytogenetic findings

Conventional cytogenetic analysis was performed on peripheral blood using unstimulated cultures. Twenty metaphases were scored and analyzed. The modal karyotype was 47,XY,del(7)(q21),+8,t(9;22)(q34;q11.2). The abnormal clone therefore demonstrated t(9;22)(q34;q11.2), with formation of the Philadelphia chromosome, trisomy 8, and del(7)(q21). All 20 metaphases analyzed demonstrated the abnormal clone. Additional cytogenetic abnormalities are recognized during CML progression and may reflect clonal evolution accompanying advanced disease [10-12].

Molecular findings

ABL1 kinase-domain mutation analysis was performed on peripheral blood. The assay detected an ABL1 p.Phe317Leu (F317L) mutation. The reported HGVS alteration was NC_000009.12(NM_005157.6).951C>A; p.Phe317Leu. The laboratory classified the variant as likely pathogenic.

The laboratory method involved RNA extraction followed by nested reverse-transcription PCR to detect the BCR::ABL1 transcript and amplify the ABL1 kinase-domain region. Kinase-domain mutations were assessed by fluorescent nucleotide sequencing followed by bioinformatic sequence analysis.

ABL1 kinase-domain mutations are a recognized mechanism of resistance to BCR::ABL1-directed therapy and are particularly relevant in patients with inadequate response or disease progression during TKI treatment [13-15]. The F317L mutation is of particular interest in this patient because of his previous dasatinib exposure [14,15]. The presence of the mutation, however, must be interpreted alongside the history of treatment default; based on the available data, it is not possible to determine the relative contribution of biological resistance and inadequate drug exposure to disease progression.

Hematopathology

The complete blood count demonstrated marked leukocytosis, anemia, and thrombocytopenia (Table 1).

**Table 1:** Complete blood count and peripheral blood differential findings Reference values are based on Dacie and Lewis Practical Haematology, 12th edition [16]. Values are expressed in the units indicated. RBC: red blood cell, MCV: mean corpuscular volume, NA: not applicable.

Parameter	Result	Reference value
Hemoglobin	7.8 g/dL	13.0-17.0-g/dL
Hematocrit	24.3%	40%-50%
RBC count	3.33 × 10⁶/µL	4.5-5.5 x 10^6^/µL
MCV	73 fL	83-101 fL
Total leukocyte count	234.3 × 10³/µL	4.0-10.0 x 10^3^/µL
Platelet count	74 × 10³/µL	150-410 x 10^3^/µL
Blasts	64%	-
Myelocytes	3%	-
Metamyelocytes	2%	-
Neutrophils	20%	40%-80%
Lymphocytes	11%	20%-40%

Peripheral blood smear examination showed microcytic hypochromic red cells with elongated forms. There was marked leukocytosis with a left shift and a prominent circulating blast population. The blasts were large, with scant cytoplasm and pleomorphic nuclei (Figure 1).

**Figure 1:**
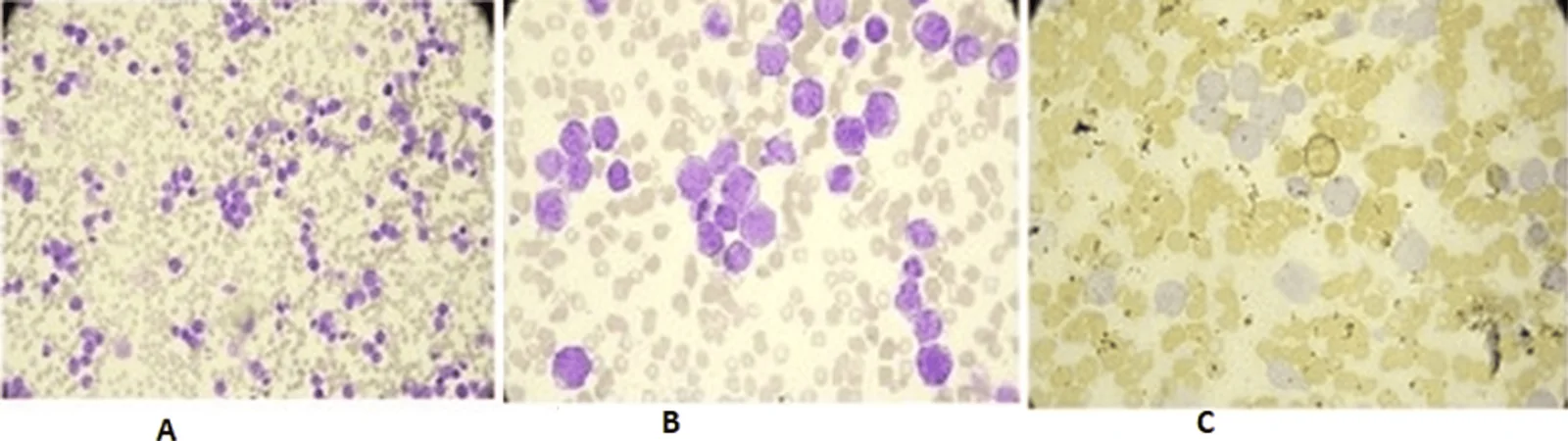
Representative peripheral blood smear (A) Leishman stain (10×) showing marked leukocytosis with a left shift and prominent circulating blasts. (B) Leishman stain (40×) showing large blasts with scant cytoplasm and pleomorphic nuclei. (C) Myeloperoxidase (MPO) stain (40×) showing negative staining in blasts.

Flow cytometric immunophenotyping

Flow cytometric immunophenotyping was performed on peripheral blood using an acute leukemia comprehensive characterization panel. An abnormal population was identified in the progenitor region, characterized by dim CD45 expression and low side scatter. This population constituted approximately 82% of all viable events. The blast population demonstrated an immature T-lineage phenotype, with cytoplasmic CD3 and CD7 positivity, dim CD5 expression, absence of surface CD3, CD1a, CD4, and CD8, together with expression of CD13 and CD33 and variable CD117. MPO was negative. B-lineage markers were absent. This profile was interpreted as early T-cell precursor lymphoblastic leukemia [8] (Table 2).

**Table 2:** Flow cytometric immunophenotypic findings CD: cluster of differentiation, HLA-DR: human leukocyte antigen-DR, TCR-γδ: T-cell receptor gamma-delta, MPO: myeloperoxidase. Dim: decreased intensity of expression, moderate: intermediate intensity of expression, variable: variable intensity of expression, positive: expression detected, negative: expression not detected.

S. no.	Category	Markers	Remarks
1	Moderate	CD34, HLA-DR, CD123, CD7, CD13, CD33	Moderate expression
2	Variable	CD56, CD117	Variable expression
3	Negative	CD19, CD10, CD20, CD22, CD1a, CD2, surface CD3, CD4, CD8, TCR-γδ, CD14, CD15, CD64, MPO	Negative
4	Positive	CD45, CD34, HLA-DR, CD38, CD123, cytoplasmic CD3, CD5, CD7, CD56, CD13, CD33, CD117	Positive expression
5	Dim	CD45, CD38, cytoplasmic CD3, CD5	Dim expression

## Discussion

Integrated diagnosis

The final diagnosis was CML in blast phase with ETP lymphoblastic differentiation, associated with an ABL1 F317L kinase-domain mutation and additional cytogenetic abnormalities, including trisomy 8 and del(7q).

The established history of CML in chronic phase is particularly important because BCR::ABL1-positive ETP-ALL can closely resemble CML blast phase. In patients without a previous diagnosis of CML, demonstration of BCR::ABL1 in both mature myeloid cells and blasts may help distinguish CML blast phase from de novo Philadelphia chromosome-positive ETP-ALL [9].

ETP phenotype in CML blast phase

CML blast phase results from progression of the CML clone, with acquisition of additional genetic abnormalities and changes in differentiation. Most CML blast-phase cases show myeloid differentiation, while approximately 20%-30% show lymphoblastic differentiation, predominantly of precursor B-cell lineage. T-cell and natural killer-cell differentiation are uncommon [4,9].

ETP-ALL is characterized by an immature T-lineage phenotype, including cytoplasmic CD3 and CD7 expression, absent or weak CD5 expression, absence of CD1a and CD8, and expression of stem-cell or myeloid-associated antigens [8]. The blasts in the present case expressed cytoplasmic CD3, CD7, and dim CD5, with absence of surface CD3, CD1a, CD4, and CD8. They also expressed CD13, CD33, and variable CD117, while MPO was negative. These findings were consistent with an ETP phenotype.

The immunophenotype was similar to that reported by Shuyu et al. in three patients with CML blast phase who presented with an ETP-ALL phenotype [3]. The presence of ETP differentiation in a patient with established CML therefore represents a rare pattern of blast transformation.

Distinguishing CML blast phase from de novo Ph-positive ETP-ALL

The major diagnostic challenge is distinguishing CML blast phase with ETP differentiation from de novo Philadelphia chromosome-positive ETP-ALL. The presence of BCR::ABL1 alone does not completely resolve this distinction because BCR::ABL1 can occur in both CML and acute lymphoblastic leukemia [9]. In the present case, the patient had a documented history of CML in chronic phase before the emergence of the ETP blast population. This clinical history supports transformation of the pre-existing CML clone.

Historical reports have described immature T-cell differentiation in Ph-positive CML blast crisis [4-6]. Senapati et al. subsequently reported ETP-type lymphoid blast crisis as a rare transformation pattern of CML [9]. More recently, Shuyu et al. reported three patients with CML blast phase presenting with an ETP-ALL phenotype and emphasized the diagnostic importance of integrating clinical, immunophenotypic, and molecular findings [3].

ABL1 F317L mutation and dasatinib

The ABL1 F317L kinase-domain mutation represents an important feature of this case. ABL1 kinase-domain mutations are recognized mechanisms of resistance to BCR::ABL1-directed TKI therapy and may be associated with disease progression [13-15]. The F317L mutation is particularly relevant in this patient because of his previous dasatinib exposure. However, the patient was also reported to be a frequent treatment defaulter. Therefore, both kinase-domain-mediated resistance and inadequate TKI exposure may have contributed to disease progression.

The available data do not include serial BCR::ABL1 transcript levels, mutation burden, duration of treatment interruptions, or documented molecular response before blast transformation. Therefore, the F317L mutation cannot be considered the sole cause of progression.

Cytogenetic clonal evolution

The cytogenetic findings provide additional evidence of disease evolution. The karyotype was 47,XY,del(7)(q21),+8,t(9;22)(q34;q11.2).

The Philadelphia chromosome represents the defining genetic abnormality of CML. Trisomy 8 and del(7q) were additional chromosomal abnormalities identified in the leukemic clone. Additional chromosomal abnormalities are recognized during CML progression and are associated with clonal evolution and an increased risk of advanced disease [10-12]. Management of blast-phase CML may include consideration of allogeneic hematopoietic stem-cell transplantation in appropriate clinical settings [16]. Additional chromosomal abnormalities have also been associated with an increased risk of disease progression [17].

Cytogenetic studies of CML blast phase have demonstrated genetic heterogeneity, with additional chromosomal abnormalities frequently accompanying transformation [10-12]. The acquisition of additional genetic abnormalities is recognized as part of the biological progression of CML to advanced phases [18]. In the present case, all 20 metaphases analyzed carried the reported abnormality, indicating that the abnormal clone was present in all cytogenetically evaluable metaphases. Additional cytogenetic abnormalities, including major-route abnormalities such as trisomy 8, have also been associated with adverse disease behavior and progression in CML [19]. Because the original chronic-phase cytogenetic result was unavailable, the exact timing of acquisition of trisomy 8 and del(7q) cannot be determined.

Integrated biological interpretation

This case demonstrates progression from established CML in chronic phase to blast phase with ETP lymphoblastic differentiation, accompanied by an ABL1 F317L kinase-domain mutation and additional cytogenetic abnormalities, including trisomy 8 and del(7q).

The findings illustrate the phenotypic and genetic evolution that can occur during CML progression. However, the exact chronological sequence of the molecular and cytogenetic events cannot be established from the available blast-phase evaluation.

Comparison with published literature

Early T-cell differentiation in CML blast crisis has been recognized for several decades. Cervantes et al. reported early T-cell features in Ph-positive CML blast crisis in 1985 [6]. Gramatzki et al. subsequently described early T-cell differentiation in CML blast crisis, supporting the potential for multilineage differentiation of the CML clone [8]. Senapati et al. later reported ETP lymphoid blast crisis as a rare transformation pattern of CML [9]. More recently, Shuyu et al. reported three patients with CML blast phase presenting with an ETP-ALL phenotype [3].

The present case adds to this limited literature by demonstrating ETP lymphoblastic differentiation together with an ABL1 F317L kinase-domain mutation and additional cytogenetic abnormalities, including trisomy 8 and del(7q), in a patient with established CML in chronic phase.

Clinical significance

CML blast phase is an aggressive stage of disease requiring assessment of blast lineage, BCR::ABL1 status, kinase-domain mutations, and additional genetic abnormalities [1,2].

The identification of an ETP phenotype has diagnostic and therapeutic implications because ETP-ALL represents an immature T-lineage leukemia [8]. In CML blast phase, treatment requires consideration of both the BCR::ABL1-positive clone and the acute blast population, with allogeneic hematopoietic stem-cell transplantation considered in appropriate patients [1,3,16]. Current and established CML management recommendations emphasize appropriate TKI therapy, response monitoring, and consideration of transplantation in advanced disease [20].

The identification of F317L is particularly relevant in a patient with previous dasatinib exposure because kinase-domain mutations may influence TKI selection [13-15]. No subsequent treatment, molecular response, transplantation, or survival information was available for inclusion in this report. Accordingly, no conclusions regarding treatment response or clinical outcome are drawn.

Limitations

The main limitation of this case is the absence of longitudinal molecular and cytogenetic data from the chronic phase. Quantitative BCR::ABL1 molecular response data, serial mutation analysis, and follow-up information after blast-phase diagnosis were also unavailable.

Therefore, although the findings strongly support CML blast phase with ETP differentiation, the exact chronological sequence of acquisition of the ABL1 F317L mutation, trisomy 8, and del(7q) cannot be established.

## Conclusions

This case represents a rare ETP lymphoblastic blast phase of CML in a 35-year-old man with established CML in chronic phase who had been treated with dasatinib and was reported to be a frequent treatment defaulter. The blast population demonstrated an ETP immunophenotype, including cytoplasmic CD3 and CD7 expression, dim CD5, CD13/CD33 coexpression, and absence of surface CD3, CD1a, CD4, CD8, and MPO. Cytogenetic analysis demonstrated 47,XY,del(7)(q21),+8,t(9;22)(q34;q11.2), establishing the presence of the Philadelphia chromosome with additional cytogenetic abnormalities. Molecular testing identified the ABL1 p.Phe317Leu (F317L) mutation.

The case underscores the importance of an integrated diagnostic approach when an immature T-cell leukemia is encountered in a patient with CML. Recognition of this rare form of blast transformation is essential for accurate classification, assessment of potential TKI resistance, and appropriate clinical management.
